# Evaluation of the remineralization potential of different bioactive glass varnishes on white spot lesions: an in vitro study

**DOI:** 10.1186/s12903-025-06665-0

**Published:** 2025-07-31

**Authors:** M. M. Sleem, El-Sayed Gad Eid, A. M. Abdelghany, D. S. Farahat

**Affiliations:** 1 Department of Dental Biomaterials, Faculty of Dentistry, Mansoura University, El-Gomhouria street, Mansoura, 35516 Egypt; 2 Spectroscopy Department, Physics Research Institute, National Research Centre, 33 Elbehouth street, Dokki, Giza, 12311 Egypt

**Keywords:** Enamel remineralization, Bioactive glass, Fluoride, Nanosilver, EDX, SEM, Microhardness

## Abstract

**Background:**

White spot lesions represent the first clinical sign of dental caries and can be reversed using various remineralizing agents. This study aimed to synthesize different bioactive glass varnishes and assess their remineralizing effects on white spot lesions compared to fluoride varnish and a synthesized nanosilver fluoride varnish.

**Methods:**

Seventy-two extracted human teeth were used. The teeth were divided into seven groups (*n* = 10): Group A (Artificial saliva, negative control), Group B (Fluoride varnish), Group C (Bioactive glass varnish), Group D (Fluoride-containing bioactive glass varnish), Group E (Nanosilver-containing bioactive glass varnish), Group F (Nanosilver fluoride varnish), and Group G (Nanosilver varnish). The different varnishes were synthesized and then characterized via transmission electron microscopy and UV-vis spectroscopy. Artificial caries were induced in the specimens by immersion in a demineralizing solution for 4 days. Each specimen was analyzed at baseline and in the demineralized state and after two weeks of varnish application by Energy Dispersive X-ray analysis and Vickers micro-hardness tester. One representative specimen from each group, one specimen at baseline, and one demineralized specimen were examined using scanning electron microscopy. Statistical analysis was conducted using one-way analysis of variance (ANOVA) and repeated measures ANOVA followed by Tukey’s post hoc test.

**Results:**

The nanosilver-containing bioactive glass group (E) presented the highest mineral gain percentage, followed by fluoride-containing bioactive glass (D) and nanosilver fluoride (F) groups, with insignificant differences between groups D and F. Groups D, E, and F showed the highest hardness recovery percentages, with insignificant differences among them. Scanning electron microscopy revealed the development of new crystals in these groups.

**Conclusions:**

Bioactive glass varnish is a promising remineralizing agent with efficacy comparable to fluoride varnish. The fluoride-containing and nanosilver-containing bioactive glass varnishes had a higher remineralizing potential compared to the fluoride and bioactive glass varnishes and nearly the same remineralizing effect of the nanosilver fluoride varnish.

## Background

Dental caries is the most common chronic infectious disease worldwide and the leading cause of dental pain. It involves progressive bacterial breakdown of tooth structure, influenced by the host, time, diet, and microorganisms. The process involves continuous cycles of demineralization and remineralization, which ultimately dictate whether the condition worsens or is reversed [1, 2]. The earliest phase of enamel demineralization is known as white spot lesions (WSLs) which are active non-cavitated lesions that can be reversed and repaired by remineralization process [3]. Remineralization is the process by which minerals lost during demineralization are replenished, serving as a natural part of the dynamic caries cycle [4].

With the current understanding of dental caries, modern caries treatment has transitioned from conventional surgical methods to a more conservative approach, aligning with the principles of minimal intervention dentistry [5]. Fluoride has been considered the principal treatment for preventing mineral loss from enamel [6]. It reduces tooth dissolution and promotes remineralization by facilitating the deposition of fluorapatite (FHA) on affected tooth surfaces or inducing the transformation of other calcium phosphate phases into FHA. This formation of FHA decreases the solubility of enamel and dentin [7]. Fluoride varnish is the preferred method for delivering topical fluoride due to its prolonged adherence to tooth surfaces, allowing it to function as a sustained fluoride reservoir [8]. However, its ability to enhance remineralization relies on the presence of calcium and phosphate ions [9]. As a result, there is a considerable demand for the development of remineralization systems that can either enhance or potentially substitute fluoride in terms of effectiveness [10].

Bioactive glass (BAG) materials possess a unique ability to replicate the body’s natural mineralization processes [11]. When in contact with body fluids, bioactive glass (BAG) releases calcium ions (Ca²⁺) and phosphate ions (PO₄³⁻), which interact to form amorphous calcium phosphate with an increase in pH. This subsequently crystallizes into hydroxyapatite (HA) [12, 13]. Bioactive glass typically contains silicon, phosphorus, calcium, sodium, and potassium. Additionally, various biologically active ions, such as zinc, silver, strontium, fluorine, iron, and copper, have been added to enhance its anti-inflammatory and antimicrobial properties [14]. The incorporation of fluoride into bioactive glass structure increases remineralization activity under acidic conditions. Fluoridated bioactive glass functions in two ways: it releases essential ions that support remineralization and provides fluoride ions that contribute to the formation of FHA [15].

Silver nanoparticles (AgNPs) have attracted significant attention due to their unique properties, such as a high surface area, antibacterial effects, controlled release of Ag^+^ ions, and biocompatibility [16]. AgNPs have been incorporated into various dental materials, adhesives, and implants to aid in caries arrest, inhibit biofilm formation, and support osteogenic induction [17]. AgNPs have also been incorporated into bioactive glass, demonstrating superior bactericidal properties compared to silver-free counterparts [18]. There is limited research assessing the remineralizing effects of incorporating silver nanoparticles (AgNPs) into bioactive glass for the treatment of white spot lesions.

When combined with silver nanoparticles, fluoride may exhibit a synergistic effect that enhances apatite crystal formation [19, 20]. Nanosilver fluoride (NSF) has gained increased attention as a replacement for silver diamine fluoride (SDF) due to its comparable effect against caries-producing bacteria without staining of dental mineralized tissue [21]. Juárez-López et al. [22] found that the combined effect of silver nanoparticles and fluoride varnish on remineralization in primary teeth was comparable to that of SDF. Nano-silver fluoride enhances enamel hardness and resistance to acid attack through a dual mechanism involving the formation of acid-resistant FHA and the infiltration and precipitation of nanosilver particles within demineralized surface pores [23].

This study aimed to synthesize different bioactive glass varnishes (bioactive glass, fluoride-containing bioactive glass and nanosilver-containing bioactive glass varnishes) and assess their remineralizing effects on white spot lesions compared to fluoride varnish and a synthesized nanosilver fluoride varnish. The null hypothesis of this study assumed that there would be no significant difference in the remineralizing effects of different bioactive glass varnishes, nanosilver fluoride varnish and fluoride varnish on white spot lesions.

## Materials and methods

This study was approved by the Research Ethics Committee of the Faculty of Dentistry at Mansoura University (Approval number A0108023DM).

### Materials

In this study, sodium-carboxymethyl cellulose and sodium fluoride were obtained from Merck (Darmstadt, Germany), while silicon dioxide, calcium carbonate, sodium carbonate, ammonium dihydrogen orthophosphate, and silver nitrate were sourced from Sigma Aldrich (St Louis, Missouri, USA). Fluor Protector S (Ivoclar Vivadent AG, Schaan, Liechtenstein) served as the fluoride varnish.

### Sample size calculation

The sample size was calculated based on a prior study by Zhang et al. [24] This indicated that a minimum of 10 samples per group were required, when the mean ± standard deviation of microhardness in the 8% BAG group was 247.3 ± 4.51 while difference with other group was 6, with 2.18 when the power was 90% & type I error probability was 0.05. The independent t-test was performed by using P.S. power 3.1.6.

### Teeth selection

Seventy-two sound human permanent incisors and canines, extracted due to periodontal disease, were collected from the oral surgery clinic at the Faculty of Dentistry, Mansoura University, in accordance with ethics committee regulations. The teeth were cleaned using an ultrasonic scaler, a rubber cup, and pumice. The teeth were disinfected in 0.5% chloramine-T for 24 h and kept in distilled water until use. The teeth were examined via a stereomicroscope at 40x magnification (Kyowa Optical, SDZ-TR-PL, Japan). Any teeth exhibiting discoloration, hypoplastic lesions, enamel cracks, fractures, or other developmental anomalies were excluded from the study.

### Grouping of the teeth

Specimens were randomly divided into seven groups (*n* = 10) according to the type of treatment:

Group A: Artificial saliva (negative control).

Group B: Fluoride varnish.

Group C: Bioactive glass varnish.

Group D: Fluoride-containing bioactive glass varnish.

Group E: Nanosilver-containing bioactive glass varnish.

Group F: Nanosilver fluoride varnish.

Group G: Nanosilver varnish.

The additional two specimens were used for SEM imaging at baseline and in the demineralized state.

### Bioactive glass powder synthesis

Bioactive glass powder with a nominal composition of 45SiO₂-24.5Na₂O-24.5CaO-6P₂O₅ (wt%) was prepared via the melt-quenching method using silicon oxide, calcium carbonate, sodium carbonate, and ammonium dihydrogen phosphate as precursor materials. The calculated batch was precisely weighed and homogenized using an agate mortar, following the method of Menazea et al. [25] The mixed powder was placed in a high-purity alumina crucible and heated in a high-temperature electric furnace (Nabertherm GmbH, Lilienthal, Germany) to 1300 °C through controlled heating to facilitate carbonate decomposition and melt homogenization. The molten glass was held at this temperature for 2 h to ensure complete melting and uniformity before being poured onto a preheated metal plate. The obtained glass (50 g) was subjected to planetary ball milling at 1000 rpm, balancing impact energy and thermal effects. To prepare the fluoride-containing bioactive glass variant, sodium fluoride (5 mol% substitution of CaO) was incorporated into the composition.

### Nanosilver particles synthesis

Colloidal silver nanoparticles were synthesized via a green eco-friendly approach using mint extract as a natural reducing and stabilizing agent. Fresh mint leaves were washed and boiled in deionized water for 10–15 min to obtain the extract, which was then filtered and centrifuged to remove solid residues. A 0.1 M silver nitrate (AgNO₃) solution was prepared separately in deionized water, and the mint extract was added dropwise under constant stirring at room temperature using a magnetic stirrer (MSH-30D, Wisd Laboratory Instruments, Daihan Scientific Co., Seoul, South Korea). The bioactive compounds in the mint extract such as polyphenols, flavonoids, and terpenoids facilitated the reduction of Ag⁺ to Ag⁰, as indicated by a shift from colorless to yellowish-brown color, following the method of Waly et al. [26] The reaction mixture was stirred for 1–2 h and kept in the dark overnight to ensure complete reduction. The synthesized nanoparticles were isolated by centrifugation, rinsed with deionized water, dried, and kept in the dark until further use.

### Preparation of varnishes

A 5% w/v solution of sodium carboxymethyl cellulose (Na-CMC) was made by dissolving the polymer in deionized water under magnetic stirring until a homogeneous mixture was obtained which was used to formulate the five synthetic varnishes. Bioactive glass and fluoride-containing bioactive glass powders were ultrasonically mixed with the Na-CMC solution at a 60:40 ratio to formulate the two varnishes. For the nanosilver-containing glass varnish, a suspension of silver nanoparticles (AgNPs, 0.1% w/v of silver) and bioactive glass powder were incorporated into the Na-CMC solution at a 60:40 ratio. In the case of the nanosilver fluoride varnish, sodium fluoride (NaF, 5% w/v) was dissolved in deionized water before being added to the Na-CMC solution containing AgNPs (AgNPs, 0.1% w/v of silver). Additionally, a nanosilver varnish was prepared by directly mixing a stable AgNPs suspension (0.1% w/v of silver) into the 5% w/v Na-CMC solution, followed by stirring to ensure uniform dispersion. All the varnishes were homogenized using ultrasonication (Sonics Vibra-Cell, Sonics & Materials, Inc., Newtown, CT, USA), packed in opaque containers and stored in a cool, dry place.

### Characterization of the varnishes

#### Transmission electron microscopy (TEM)

A transmission electron microscope (Talos L120C G2, Thermo Fisher Scientific, USA) was utilized at 110 kV to assess the particle size and morphology of the synthesized powders. A drop of the bioactive glass, nanosilver fluoride and nano-silver varnishes were individually gradually evaporated at room temperature on a 400-mesh copper grid, which was then covered by a carbon support film. Selected area electron diffraction (SAED) was conducted to evaluate the crystallinity of the varnishes.

#### UV-vis spectroscopy

UV–visible spectroscopy was conducted utilizing a UV/vis spectrometer (T80 + UV/vis spectrometer, PG Instruments, UK). Measurements were carried out in 1.0 cm stoppered silica cuvettes using a dual-beam mode with a spectral bandwidth of 1.0 nm, covering the wavelength range of 190–1100 nm.

### Teeth preparation

The root portion of each tooth was removed by a low-speed diamond saw (Isomet; Buehler, Lake Bluff, IL, USA) while being cooled with running water. The coronal part was then mounted in acrylic resin (Acrostone, Egypt) molds with the labial surface exposed. The labial surface was polished with finishing and polishing disks (TOR VM 1.071, TOR VM Ltd, Moscow, Russia) and a low-speed contra-angle handpiece (ROSE 201CA, China) at a speed lower than 30,000 according to the manufacturer’s instructions. A 4 × 4 mm window on the enamel surface was created by coating the remaining surface with nail varnish (EVA cosmetics, Egypt) and the samples were stored in deionized water until needed.

### Preparation of artificial caries lesions

Demineralization was induced by immersing the specimens in a solution that contained 2.2 mM calcium chloride (CaCl_2_), 2.2 mM sodium dihydrogen orthophosphate dehydrate (NaH_2_PO_4_), and 0.05 M acetic acid. The pH was adjusted to 4.4 with 1 M potassium hydroxide [27]. Each specimen was individually immersed daily in a freshly prepared demineralizing solution for 96 h to develop a uniform white spot lesion [28]. The specimens were subsequently washed and kept in deionized water [29].

### Application of the remineralizing varnishes to enamel specimens

Each sample was treated with the intended varnish using a microbrush for one minute. Extra varnish was removed using gentle air pressure, then each sample was rinsed with deionized water and stored in freshly prepared artificial saliva for 14 days. The artificial saliva was changed for each specimen once every 24 h. For the control group, specimens were only washed with deionized water and then stored in artificial saliva. The artificial saliva was made following the method by Ten Cate and Duijsters, consisting of 1.5mM CaCl_2_, 0.9mM NaH_2_PO_4_, and 0.15 M KCl with a pH of 7.0 [30].

### Assessment of enamel remineralization potential of the different varnishes

#### Assessment of surface micromorphology

One specimen was randomly selected from each group and evaluated for structural changes using a scanning electron microscope (SEM) (Quanta FEG 250, FEI Company, Hillsboro, Oregon, USA) after varnish application and storage in artificial saliva for two weeks. A specimen at baseline (before induction of carious lesions) and a demineralized specimen were also scanned at 4000x magnification and a voltage of 30 kV. A thin layer of gold was deposited on the samples using a gold sputter coater (Model DSR1, VacCoat, UK) with a power of 700 W, operating at 110 V and 50/60 Hz, prior to imaging using the scanning electron microscope.

#### Energy dispersive X-ray (EDX) analysis of surface mineral content

The calcium and phosphorus mineral content on the surface of each specimen was examined using an energy-dispersive X-ray spectroscope (EDS) (Quanta FEG 250, FEI Company, Hillsboro, Oregon, USA) integrated with the environmental scanning electron microscope with an applied voltage of 20 kV then the Ca/P was calculated. The examination was performed at: T_0_: Baseline (sound specimen), T_1_: after carious lesion induction, and T_2_: after varnish application and storage in artificial saliva for two weeks. The mineral gain percentage was determined by the formula:

$$\:\%\:\Delta\:\frac{Ca}{P}=\frac{\frac{Ca}{P}at\:T2-\frac{Ca}{P}at\:T1}{\frac{Ca}{P}at\:T2}\times\:100$$ [31]

#### Assessment of surface microhardness

Surface microhardness testing serves as an indirect method for assessing mineral content, with higher calcium and phosphorus levels correlating with increased hardness values [32]. The enamel surface microhardness was evaluated by Vickers microhardness tester (Jinan Precision testing equipment co., Model HV-1000 LTD, China). Indentations were created on the surface using a diamond microindenter with a pyramidal shape and a 136° angle between its faces, applying a 100 g load for 10 s. The surface hardness (SH) was automatically determined by the equation: SH = 1854.4P/d^2^ where P represents the applied load (g), and d is the average length of the diagonals of the indentations (µm). The indentations were performed three times and a minimum spacing of 50 μm was maintained between consecutive indents to prevent any influence from residual stresses of adjacent impressions [33]. To avoid the influence of previous indentations, it was ensured that hardness measurements at each timepoint (baseline, post-demineralization, and post-remineralization) were performed at different locations on the sample surface within the created 4 × 4 ml window. The average was calculated and recorded for each specimen at baseline, after carious lesion induction and 14 days after application of the remineralizing varnishes [28]. The percentage of surface microhardness recovery was calculated by the following equation:

$$\:{\%}\text{S}\text{M}\text{H}\text{R}=\frac{({SH}_{2}-{SH}_{1})}{({SH}_{0}-{SH}_{1})}\times\:100\%$$ [34]

where %SMHR is the percentage of surface microhardness recovery, SH_0_ is the surface microhardness at baseline, SH_1_ is the surface microhardness after demineralization and SH_2_ is the surface microhardness 14 days post varnish application.

### Statistical analysis

Statistical analysis was conducted using SPSS 20^®^ software. The normality of the data was assessed using Shapiro-Wilk and Kolmogorov normality test, which confirmed that all data followed a normal distribution. One-way analysis of variance ANOVA test was conducted to compare between more than 2 groups, followed by Tukey’s post hoc test for multiple comparisons. Repeated measures ANOVA test was used to compare the results at three different times (T_0_, T_1_, T_2_) in each followed by Tukey’s post hoc test for multiple comparisons. The significance level was set at *P* < 0.05.

## Results

### Characterization of the synthesized varnishes

#### Transmission electron microscopy (TEM)

**Fig. 1:**
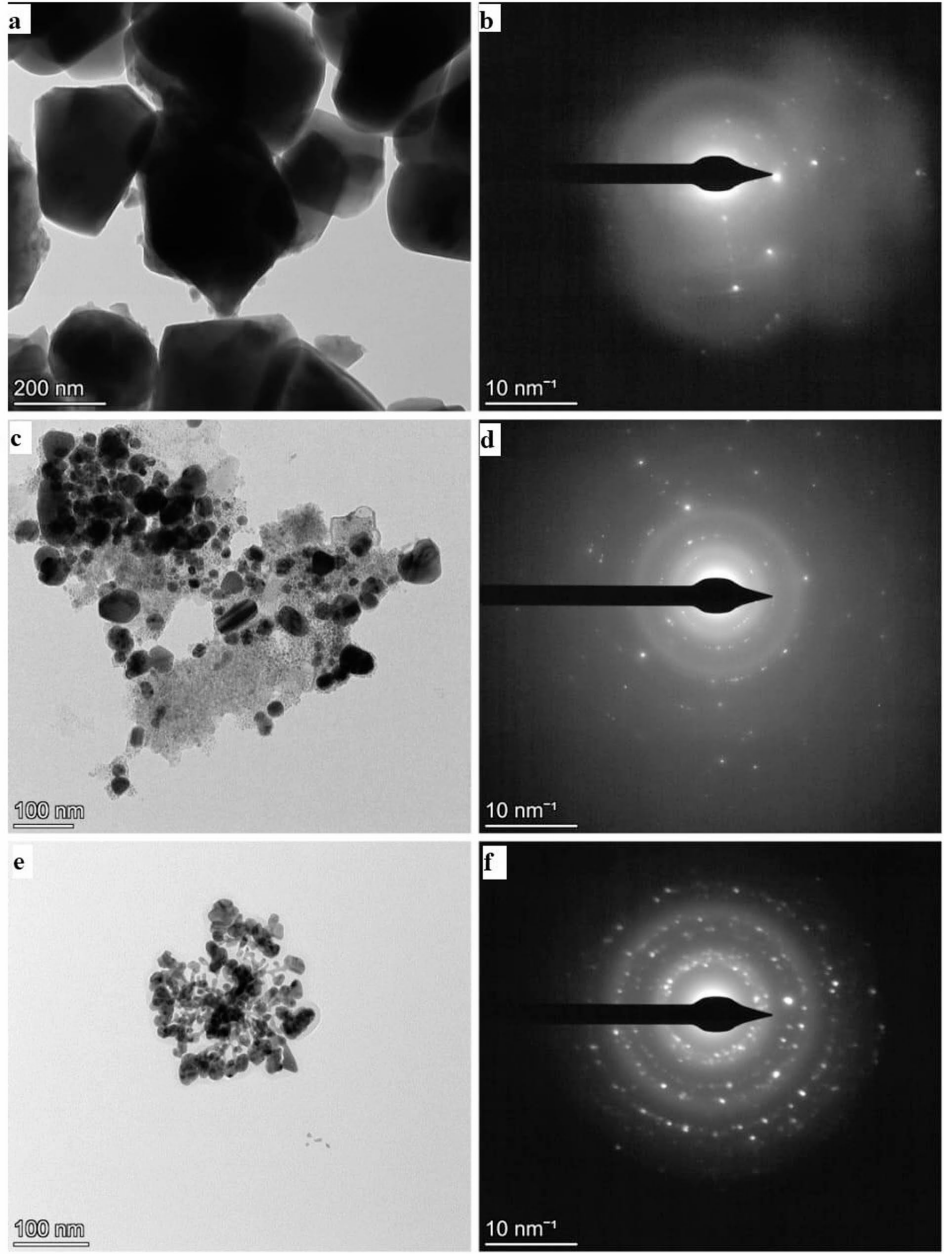
**(a)** TEM image of bioactive glass varnish, **(b)** SAED of bioactive glass varnish, **(c)** TEM image of nanosilver fluoride varnish **(d)** SAED of nanosilver fluoride varnish **(e)** TEM image of nanosilver varnish **(f)** SAED of nanosilver varnish

The bioactive glass varnish contained irregular polyhedral glass particles, varying in size from 50 nm to 300 nm (Fig. 1a). The nanosilver fluoride varnish contained densely dispersed spherical silver nanoparticles, ranging in size from 10 nm to 50 nm, along with smaller, low-contrast features corresponding to sodium fluoride distributed throughout the polymer matrix (Fig. 1c). Analysis of the nano-silver varnish confirmed the presence of spherical nanoparticles ranging in size from 10 nm to 50 nm (Fig. 1e).

#### Selected area electron diffraction (SAED)

SAED examination of bioactive glass varnish showed diffuse rings with some distinct diffraction spots, indicating the formation of crystalline structures within the amorphous glass structure (Fig. 1b). Examination of nanosilver fluoride varnish revealed well-defined concentric rings with bright spots, characteristic of a polycrystalline structure (Fig. 1d). Finally, the nanosilver varnish showed concentric rings with superimposed bright spots (Fig. 1f).

#### UV-vis spectroscopy

**Fig. 2:**
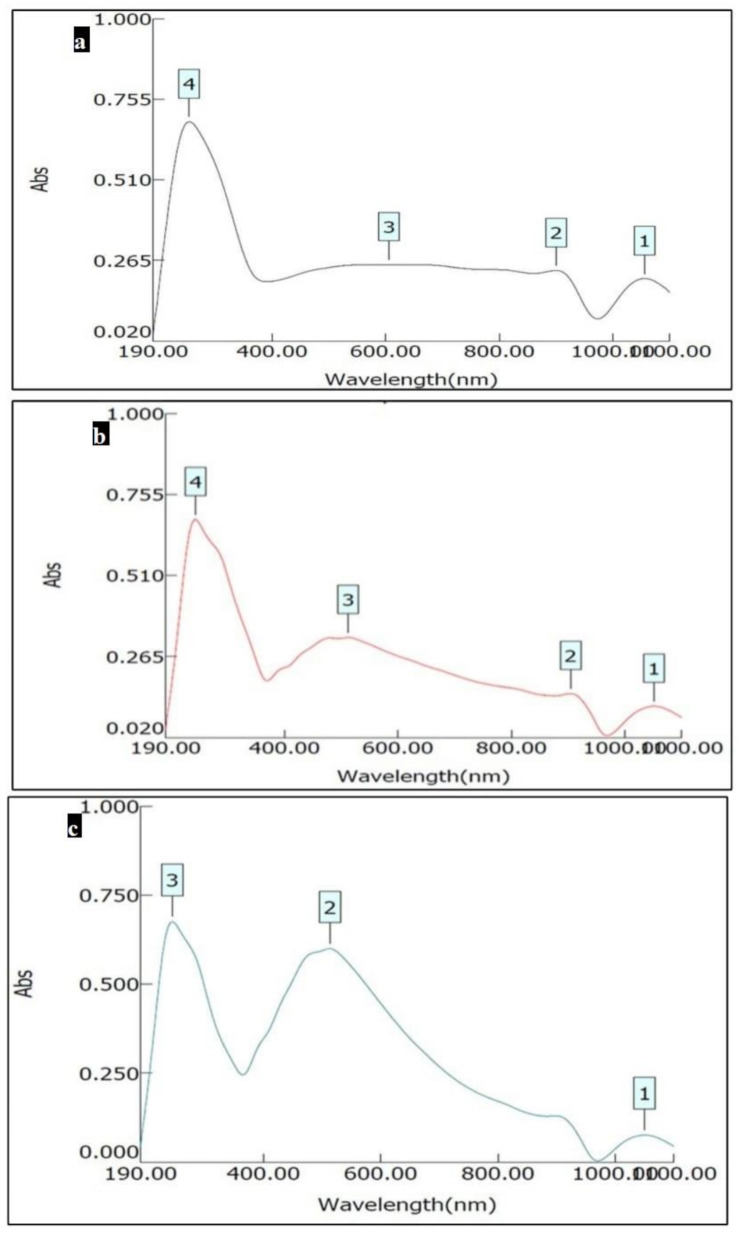
UV-vis spectroscopic analysis of **(a)** Bioactive glass varnish **(b)** Nanosilver fluoride varnish **(c)** Nanosilver varnish

Bioactive glass varnish showed sharp absorbance peaks at 254 nm (Fig. 2a). Nanosilver fluoride varnish exhibited absorbance peaks at 243 and 512 nm (Fig. 2b). Nanosilver varnish represented peaks at 244 and 513 nm (Fig. 2c).

### Evaluation of the remineralization potential of the varnishes

#### Morphological analysis

**Fig. 3:**
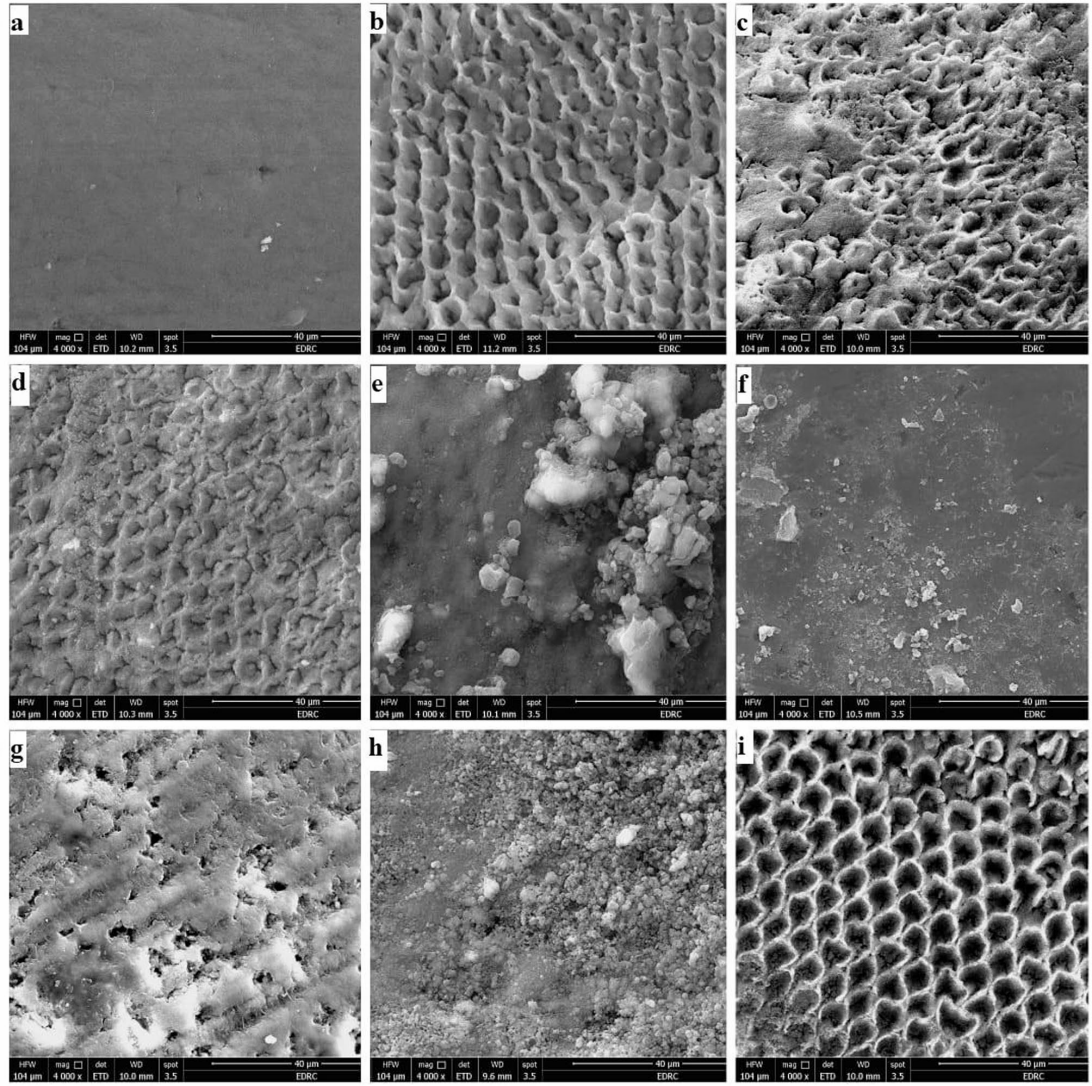
SEM micrographs of the enamel surface of specimens **(a)** At baseline **(b)** After application of demineralizing solution **(c)** Artificial saliva group **(d)** Fluoride varnish group **(e)** Bioactive glass varnish group **(f)** Fluoride-containing bioactive glass varnish group **(g)** Nanosilver-containing bioactive glass varnish group **(h)** Nanosilver fluoride varnish group **(i)** Nanosilver varnish group

The SEM analysis of the enamel surface at baseline revealed a crystalline structure characterized by intact enamel prisms and a well-preserved interprismatic region, with small defects observed between the calcified areas (Fig. 3a). After demineralization, the SEM image of the enamel surface showed enamel prisms that were uniformly demineralized with honeycomb pattern (Fig. 3b). The artificial saliva group exhibited a porous surface suggesting limited repair of the enamel structure (Fig. 3c). The fluoride varnish group showed partial closure of prism pores with the formation of hydroxyapatite crystals (Fig. 3d). The bioactive glass varnish group exhibited clusters of crystals and closure of enamel prisms with minimal porosity (Fig. 3e). The fluoride-containing bioactive glass varnish group exhibited decreased surface porosity and the formation of a crystalline layer (Fig. 3f). The nanosilver-containing bioactive glass group displayed the formation of crystals inside enamel pores that decreased the pore size (Fig. 3g). Nanosilver fluoride group showed a layer of crystals covering enamel surface and closing surface porosities (Fig. 3h). The nanosilver varnish group showed an irregular surface with multiple pores (Fig. 3i).

#### Elemental analysis of surface mineral content

**Fig. 4:**
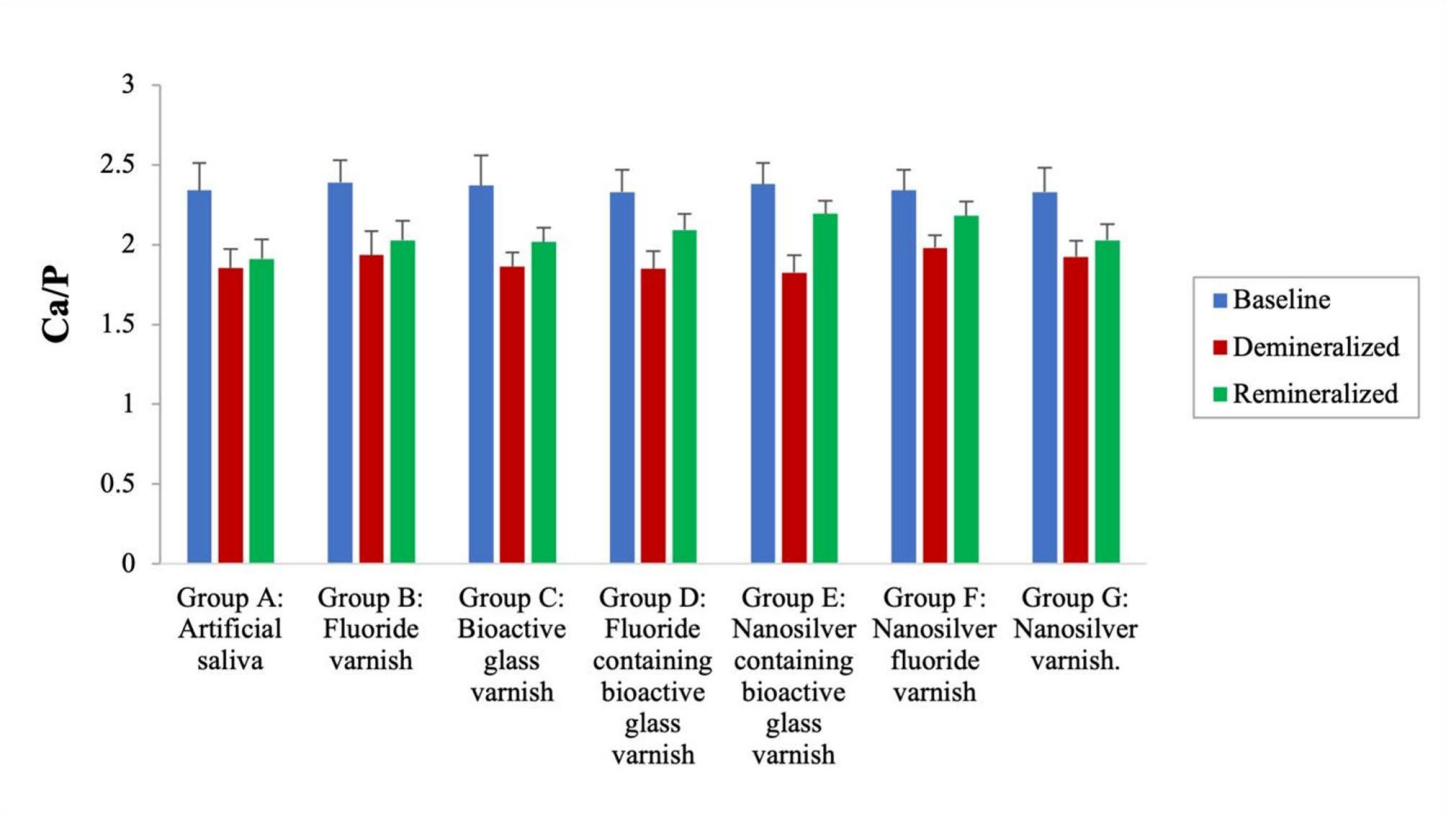
Bar chart of Ca/P ratio at baseline, after demineralization and 14 days after varnish application in different test groups

**Fig. 5:**
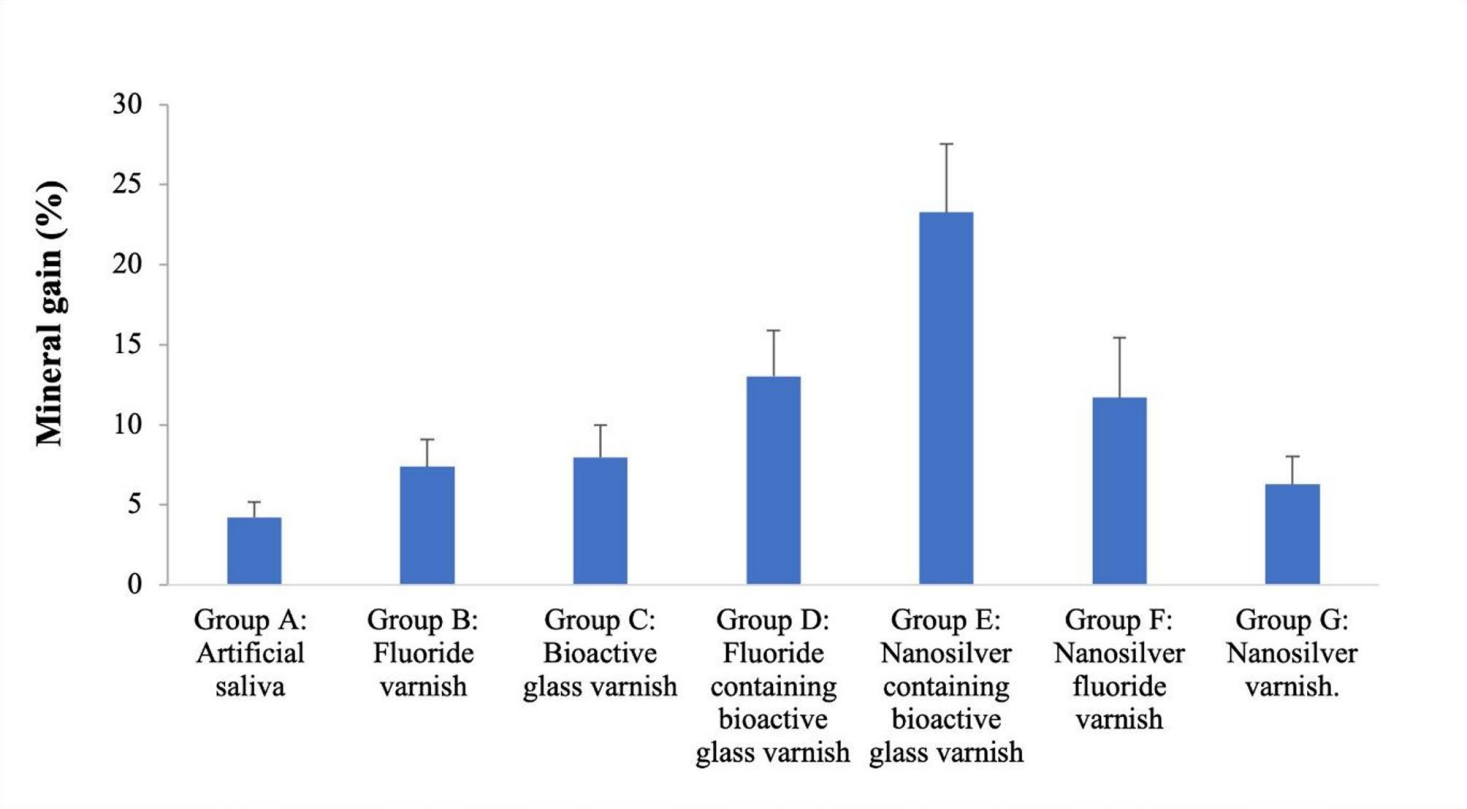
Bar chart of mineral gain percentage in different test groups

At baseline, insignificant differences were observed in the Ca/P ratio among all groups (*P* = 0.95). Following demineralization, all groups exhibited a significant reduction in the Ca/P ratio compared to that at baseline (*P* < 0.001) and insignificant differences were observed among the groups, except between Group E and Group F (*P* = 0.02). Following the application of remineralizing agents, all groups exhibited a significant increase in the Ca/P ratio compared to the demineralized state (*P* < 0.001). Regarding mineral gain %, nanosilver-containing bioactive glass group showed the significantly highest mineral gain followed by fluoride-containing bioactive glass (D) and nanosilver fluoride (F) groups. However, no significant difference was found between groups D & F. Bioactive glass, fluoride and nanosilver groups had statistically indifferent mineral gain percentages. Artificial saliva group presented the lowest mineral gain percentage among all groups. Means and standard deviations of Ca/P ratio and mineral gain percentage are represented in Table 1; Figs. 4 and 5.

**Table 1:** Ca/P ratio (at baseline, after demineralization and 2 weeks post varnish application) and enamel mineral gain percentages of the different studied groups

Group	Baseline	After exposure to demineralizing solution	2 Weeks post varnish application	Mineral gain %
Group A: Artificial saliva	2.34 ± 0.17^Aa^	1.852 ± 0.12^Bab^	1.912 ± 0.12^Ca^	4.23 ± 0.96^a^
Group B: Fluoride varnish	2.39 ± 0.14^Aa^	1.935 ± 0.15^Bab^	2.028 ± 0.12^Cb^	7.39 ± 1.70^b^
Group C: Bioactive glass varnish	2.37 ± 0.19^Aa^	1.861 ± 0.09^Bab^	2.016 ± 0.09^Cb^	7.98 ± 1.99^b^
Group D: Fluoride-containing bioactive glass varnish	2.33 ± 0.14^Aa^	1.849 ± 0.11^Bab^	2.093 ± 0.10^Cc^	13.02 ± 2.85^c^
Group E: Nanosilver-containing bioactive glass varnish	2.38 ± 0.13^Aa^	1.824 ± 0.11^Ba^	2.194 ± 0.08^Cc^	23.27 ± 4.26^d^
Group F: Nanosilver fluoride varnish	2.34 ± 0.13^Aa^	1.98 ± 0.08^Bb^	2.181 ± 0.09^Cc^	11.70 ± 3.76^c^
Group G: Nanosilver varnish	2.33 ± 0.15^Aa^	1.924 ± 0.10^Bab^	2.027 ± 0.10^Cb^	6.29 ± 1.72^b^

*Significant difference at *P* < 0.05

Means with different superscripted lowercase letters in the same column were significantly different at *P* < 0.05

Means with different superscripted uppercase letters in the same row were significantly different at *P* < 0.05

#### Assessment of surface microhardness

**Fig. 6:**
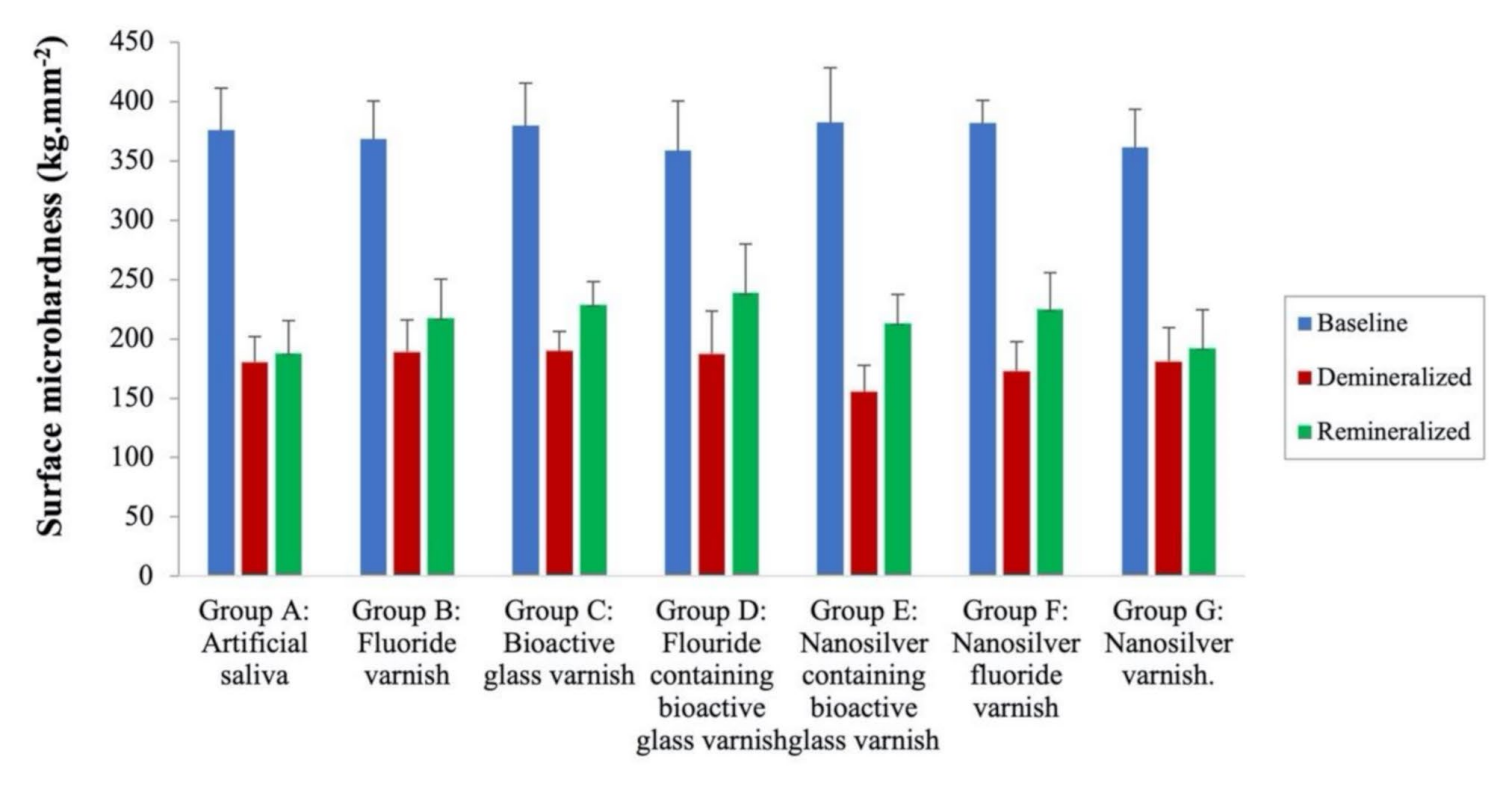
Bar chart of enamel surface microhardness at baseline, after demineralization and after remineralization in different test groups

**Fig. 7:**
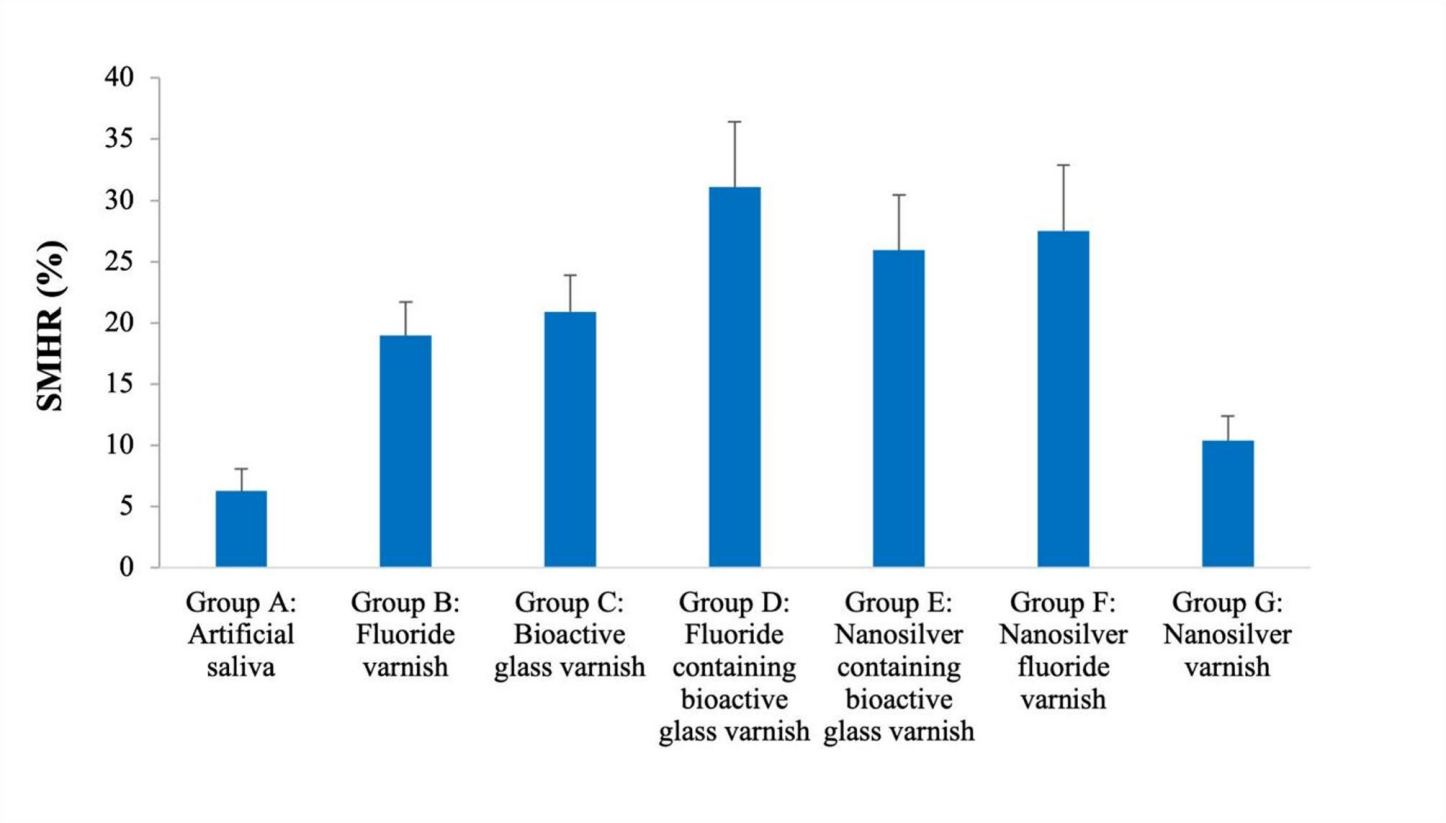
Bar chart of the percentage of surface microhardness recovery (%SMHR) in different test groups

At baseline and after demineralization, there were no significant differences in surface microhardness among all the groups (*P* = 0.61 at baseline, *P* = 0.06 after demineralization). A significant reduction in Vickers microhardness was observed in all groups after demineralization (*P* < 0.001). After the application of remineralizing varnishes, Vickers microhardness significantly increased in all groups (*P* < 0.001) except for Group A (artificial saliva) which was not significantly different from that in the demineralized state. Regarding surface microhardness recovery percentage (%SMHR), there were highly significant differences between groups (*p* < 0.001). Fluoride-containing bioactive glass, nanosilver fluoride and nanosilver-containing bioactive glass groups showed the highest SMHR% with no significant differences between them followed by bioactive glass and fluoride groups which were not significantly different. The difference between the nanosilver-containing bioactive glass and bioactive glass groups was not statistically significant. The nanosilver and artificial saliva groups showed the lowest hardness recovery percentage with no significant difference between them. Data are represented in Table 2; Figs. 6 and 7.

**Table 2:** Vickers microhardness (kg.mm^− 2^) and enamel surface microhardness recovery percentage

Group	Baseline	After exposure to demineralizing solution	2 weeks post varnish application	SMHR%
Group A: Artificial saliva	376.00 ± 35.13^Aa^	180.40 ± 21.62^Ba^	187.9 ± 27.28^Ba^	6.28 ± 1.80^a^
Group B: Fluoride varnish	368.20 ± 32.56^Aa^	188.80 ± 26.89^Ba^	217.5 ± 32.85^Ca^	18.97 ± 2.75^b^
Group C: Bioactive glass varnish	379.60 ± 36.06^Aa^	189.70 ± 16.74^Ba^	228.8 ± 19.63^Cb^	20.93 ± 2.99^bc^
Group D: Fluoride-containing bioactive glass varnish	358.70 ± 41.69^Aa^	187.40 ± 35.92^Ba^	238.6 ± 41.15^Cb^	31.10 ± 5.33^d^
Group E: Nanosilver-containing bioactive glass varnish	382.20 ± 46.21^Aa^	155.40 ± 22.35^Ba^	213.1 ± 24.29^Ca^	25.95 ± 4.49^cd^
Group F: Nanosilver fluoride varnish	381.70 ± 19.60^Aa^	172.80 ± 25.10^Ba^	224.7 ± 31.11C^ab^	27.52 ± 5.38^d^
Group G: Nanosilver varnish	361.30 ± 32.27^Aa^	180.80 ± 28.49^Ba^	192.1 ± 32.61^Ca^	10.40 ± 2.02^a^

*Significant difference at *P* < 0.05

Means with different superscripted lowercase letters in the same column were significantly different at *P* < 0.05

Means with different superscripted uppercase letters in the same row were significantly different at *P* < 0.05

## Discussion

This study aimed to synthesize different bioactive glass varnishes (bioactive glass, fluoride-containing bioactive glass and nanosilver-containing bioactive glass varnishes) and assess their remineralizing effects on white spot lesions compared to a commercially available fluoride varnish and a synthesized nanosilver fluoride varnish. There was a significant difference in the remineralizing effect of the different varnishes so the null hypothesis had to be rejected.

White spot lesions (WSLs) are areas of demineralized enamel that can progress into cavities and result from pH changes at the tooth-biofilm interface, driven by bacterial metabolism [35]. Calcium and phosphate are crucial in regulating tooth demineralization and remineralization [36]. Therefore, materials that provide calcium and phosphate can support the remineralization process, making bioactive glass a suitable choice for this study. BAG increases the pH of the oral environment by releasing calcium and phosphorus ions, which facilitate apatite formation [37]. Fluoridated bioactive glass has the capability to generate FHA which improves enamel resistance to acid attack. Numerous studies have evaluated the antibacterial effects of incorporating silver ions into bioactive glass [38, 39]. However, research on their impact on remineralization is limited. Accordingly, this study investigated the remineralizing effect of adding nanosilver particles to bioactive glass varnish.

The results of the TEM analysis in this study showed that the bioactive glass particles exhibited irregular polyhedral shapes with particle sizes varying between 50 and 300 nm, while silver nanoparticles had spherical shapes with sizes 10–50 nm. This was in accordance with the results of a study by Shi et al. [40] who reported bioactive glass particles with sizes ranging from 280 to 300 nm and Ioannidis et al. [41] who documented spherical AgNPs with diameters spanning from 20 to 50 nm. SAED examination of bioactive glass varnish showed diffuse rings which are characteristic of the amorphous nature of glassy materials [42, 43]. Bioactive glasses often crystallize during preparation or at high temperatures due to the low connectivity of disrupted silicate network. The increase in non-bridging oxygen atoms weakens silicate bonds, increasing structural mobility and promoting nucleation. Consequently, 45S5 glass is prone to bulk or surface crystallization when particle size or heat-treatment conditions change [44]. This explains the presence of crystalline structures within the amorphous glass framework. Maity et al. [45] synthesized methylcellulose-silver nanocomposite and performed SAED which revealed sharp spots within distinct diffraction ring patterns.

UV-vis spectroscopy analysis of the bioactive glass varnish exhibited sharp absorbance peaks at 254, indicating the presence of silicate glass [46]. Sharp absorbance peaks at 243 and 244 nm in nanosilver fluoride varnish and nanosilver varnish, respectively, indicate the presence of metallic silver nanoparticles (AgNPs) [47]. There were broad absorption peaks at 512 nm and 513 nm in nanosilver fluoride varnish and nanosilver varnish, respectively. This is consistent with the characteristic surface plasmon resonance peak of AgNPs, which typically appears between 400 and 500 nm, while nanoparticle agglomeration can shift the peak wavelength up to 600 nm [48].

The SEM analysis of the fluoride-containing bioactive glass varnish group demonstrated crystal formation on the enamel surface, accompanied by partial structural restoration. The nanosilver-containing bioactive glass varnish group exhibited crystal deposition within enamel porosities. Additionally, SEM imaging of specimens treated with nanosilver fluoride varnish revealed a uniform crystalline layer covering the enamel surface, with significant restoration of the interprismatic structure. Furthermore, the bioactive glass varnish group showed the formation of a new crystalline layer with small residual surface pores. Eldeeb et al. [49] reported the deposition of mineral crystals filling prism cores on fluoridated bioactive glass-treated enamel surfaces. Similarly, an SEM study by Seifi et al. [50] reported that an adhesive incorporating silver-doped bioactive glass promoted crystal formation and enamel remineralization. Also, the SEM observations of the nano-silver fluoride group in the study by Favaro et al. [51] showed newly formed crystals on the enamel surface. Likewise, Dong et al. [52] observed the development of a dense, compact crystalline layer on demineralized enamel surfaces following treatment with 45 S melt-quench bioactive glass which is in alignment with our study.

The Ca/P ratio is a key indicator of caries resistance in dental tissues, as hydroxyapatite crystals participate in the dynamic physicochemical processes of demineralization and remineralization [53]. The results of the current study showed that the Ca/P ratios at baseline were statistically similar across all groups. Following demineralization, insignificant differences were observed among the groups, except for groups E and F. This may be attributed to the age variability among the patients from whom the teeth were obtained, as age-related changes in older individuals can influence the hardness and mineral composition of sound enamel [54]. The nanosilver-containing bioactive glass group had the highest percentage of mineral gain among the test groups followed by the fluoride-containing bioactive glass group and nanosilver fluoride group. Shirkhanzadeh et al. [55] reported that silver ions strongly stimulate carbonate apatite formation by acting as nucleation sites and their release in simulated body fluids may lower the necessary calcium and phosphorus levels for apatite formation. This could explain the higher remineralization effect of the silver containing bioactive glass varnish compared to the nanosilver varnish as the bioactive glass component provides the ions needed to form the apatite crystals [12].

On the other hand, the improved mineral gain in the fluoride-containing bioactive glass group could be attributed to the combined effect of bioavailable calcium, phosphorus and fluoride. Manzoor et al. [56] found that fluoride-containing bioactive glass exhibited a more pronounced remineralization effect on artificially induced dentine caries compared to non-fluoridated bioactive glass. Also, the results of a study by Shen et al. [57] revealed that incorporating calcium and phosphate into fluoride dentifrices significantly improved the mineral content of white spot lesions compared to fluoride-only formulations. The results of a study by Mohamed et al. [58] similarly revealed that remineralization using a nanosilver fluoride varnish resulted in a higher Ca/P ratio than the fluoride group.

Bioactive glass, fluoride, and nanosilver varnishes did not significantly differ, but demonstrated a significantly higher percentage of mineral gain compared to artificial saliva. These results are in accordance with the results of a study by Kölüş et al. [59] who concluded that bioglass group had a higher Ca/P ratio than fluoride group but statistically, they were similar. Also, Rahman et al. [60] found that bioactive glass increased C/P ratio significantly compared to artificial saliva after two weeks of storage as it facilitates remineralization by providing calcium and phosphate ions, where they form a supersaturated solution that precipitates onto hard dental tissues [61].

The mechanical properties of enamel are strongly influenced by its mineral content, as hypomineralized enamel exhibits reduced surface hardness [62]. The results of the present study showed that there were no significant differences in surface microhardness among all the groups at baseline and after demineralization. After varnish application, all groups showed significant increase in enamel hardness except the negative control group. Although no statistically significant differences were observed in initial microhardness or in the demineralized state among the groups, microhardness recovery was calculated to account for these variations and enable a more precise comparison among the groups [50].

The fluoride-containing bioactive glass, nanosilver fluoride and nanosilver-containing bioactive glass varnish groups had no statistically significant differences between them and demonstrated the highest hardness recovery percentages. The nanosilver-containing bioactive glass varnish produced higher percentages of enamel hardness recovery compared to the nanosilver and bioactive glass varnishes although there wasn’t a significant difference with the later. This could be attributed to the synergistic interaction between the nanosilver and the bioactive glass. Specifically, the nanosilver may serve as nucleation sites for apatite crystal formation while the bioactive glass contributes essential calcium and phosphate ions necessary for the formation and growth of these crystals [55]. It was suggested that silver ions could infiltrate and precipitate on carious lesions increasing their surface hardness [63]. However, Favaro et al. [51] reported the low remineralization effect of a colloidal silver nanoparticles solution with the formation of precipitates, that were less concentrated and dispersed without a remineralization layer. Rodriguez et al. [64] compared the effect of incorporating bioactive glass, bioactive glass with 0.2 mol% and 0.5 mol% Ag_2_O into toothpaste, concluded that all glasses improved remineralization but the surface hardness loss% differences were insignificant.

Studies proposed that, in agents which contain high concentrations of fluoride, fluoride ions have high affinity to combine with calcium ions in the enamel which might prevent fluoride ions from penetrating the demineralized enamel surfaces. On the other hand, in agents with low fluoride concentration, like fluoride-containing bioactive glass, the calcium and phosphate ions could penetrate the enamel surfaces and remineralize the body of the lesions [65, 66]. Furthermore, the presence of low concentrations of fluoride might facilitate apatite formation, since fluoride is known to catalyze the conversion of brushite, octacalcium phosphate, and amorphous calcium phosphate to apatite [67]. Farooq et al. [68] found that fluoridated bioglass toothpaste showed higher surface micro-hardness in comparison to the fluoride toothpaste. El-Damanhoury et al. [69] found in their study that fluoridated calcium phosphosilicate group had significantly higher surface hardness than bioactive glass.

Nanosilver fluoride group has significantly higher hardness recovery than fluoride, silver and negative control groups. Favaro et al. [51] reported that the nanosilver fluoride solution resulted in a significantly greater increase in surface hardness compared to both nanosilver and fluoride treatments individually. Nozari et al. [63] found that nanosilver fluoride varnish group showed significantly higher surface microhardness than fluoride and negative control groups.

The bioactive glass and fluoride varnish groups showed no statistically significant difference in hardness recovery percentage, both exhibiting higher values than the artificial saliva group, aligning with the mineral gain percentage results. Sherif et al. [70] reported that bioactive glass exhibited greater surface microhardness compared to sodium fluoride; however, the difference was not statistically significant. Rahman et al. [60] found in their study that bioactive glass had significantly higher hardness recovery than artificial saliva. On the other hand, the nano-silver group exhibited significantly higher mineral deposition compared to the artificial saliva group but showed no significant difference in hardness recovery. This could be explained by transverse microradiography findings from another study by Aldhaian et al. [71] that found that silver promotes some remineralization primarily in the outer regions of the lesion, with limited effects on deeper parts within the lesion. In the current study, artificial saliva showed the least remineralization of enamel surface due to limited calcium and phosphate release [72, 73].

One limitation of this in vitro study is that it does not replicate the diverse conditions present in the oral cavity such as chewing, brushing, enzymes, and tongue movement that might affect the long-term retention of the varnishes on enamel surfaces. Clinical trials are needed for a better understanding of the effect of the oral conditions on the varnish wear and stability. Moreover, this study only investigated one concentration of bioactive glass varnish and used a 14-day storage period. Future research should explore different concentrations and longer storage durations to understand how the varnish works over time. Additionally, evaluating the depth of remineralization could provide more insight about the remineralizing effect of different varnishes.

## Conclusions

Bioactive glass varnish is a promising remineralizing agent with efficacy comparable to fluoride varnish. The fluoride-containing and nanosilver-containing bioactive glass varnishes had a higher remineralizing potential compared to the fluoride and bioactive glass varnishes and nearly the same remineralizing effect of the nanosilver fluoride varnish.

## Data Availability

The datasets utilized and/or analyzed in this study are available from the corresponding author upon reasonable request.

## Abbreviations

WSLs: White spot lesions
HA: Hydroxyapatite
FHA: Fluoridated hydroxyapatite
BAG: Bioactive glass
AgNPs: Silver nanoparticles
TEM: Transmission electron microscope
SAED: Selected area electron diffraction
SEM: Scanning electron microscope
EDS: Energy-dispersive X-ray spectroscope
SH: Surface microhardness
%SMHR: Percentage of surface microhardness recovery
NSF: Nanosilver fluoride
SDF: Silver diamine fluoride
